# The frequency and inter-relationship of PD-L1 expression and tumour mutational burden across multiple types of advanced solid tumours in China

**DOI:** 10.1186/s40164-020-00173-3

**Published:** 2020-08-03

**Authors:** Yanhui Chen, Yating Wang, Hongli Luo, Xue Meng, Wei Zhu, Di Wang, Hui Zeng, Henghui Zhang

**Affiliations:** 1 Institute of Infectious Diseases, Beijing Ditan Hospital, Key Laboratory of Emerging Infectious Diseases, Capital Medical University, No. 8 Jingshun East St, Beijing, 100015 China; 2 Genecast Biotechnology Co., Ltd., 35 Huayuan North Rd, Beijing, 100089 China

**Keywords:** PD-L1, TMB, PD-1, Solid tumour, Immunotherapy

## Abstract

**Background:**

PD-L1 expression and tumour mutational burden (TMB) are both associated with the responses of multiple tumours to immune checkpoint inhibitor (ICI) therapy. However, their prevalence and correlations may differ in different types of advanced solid tumours.

**Methods:**

PD-L1 expression, TMB, and PD-1^+^ Tils (tumour-infiltrating lymphocytes) infiltration and their relationships were assessed in 6668 advanced solid tumour specimens across 25 tumour types. CD8^+^ T cell infiltration was analysed in 347 NSCLC samples. The associations of these biomarkers with the therapeutic effect of PD-1 inhibitor were analysed in a cohort of NSCLC samples.

**Results:**

PD-L1 expression levels and TMB in different tumour types varied widely and their relationship was not significantly correlated in most cancer types, with only a small association across all specimens (Spearman R = 0.059). PD-1^+^ Tils infiltration was positively correlated with PD-L1 expression across all samples (Spearman R = 0.3056). However, there is no such correlation between PD-1^+^ Tils infiltration and TMB. In NSCLC samples, CD8^+^ T cell infiltration was correlated with PD-1^+^ Tils infiltration and PD-L1 expression but not with TMB (Spearman R = 0.4117, 0.2045, and 0.0007, respectively). Patients in the CR/PR group (anti-PD-1 therapy) had higher levels of PD-L1 expression, TMB, PD-1^+^ Tils, and CD8^+^ T cell infiltration, and many patients in this group exhibited concomitantly elevated levels of multiple biomarkers.

**Conclusions:**

Our results showed the PD-L1 expression status and TMB in various types of advanced solid tumours in Chinese patients and their relationships with PD-1^+^ Tils and CD8^+^ T cell infiltration, which may inform ICI treatment.

## Background

Tumour immunotherapy, especially immune checkpoint inhibitor (ICI) therapy, has been progressing rapidly. At present, not many ICIs have been approved for clinical use in China, and their indications are still few. Most ICIs and indications are still in clinical trials. However, recent studies have found that ICI therapies on some solid tumours, such as oesophageal cancer, hepatocellular carcinoma, and gastric or gastroesophageal junction (G/GEJ) adenocarcinoma, have a better efficacy in China or Asia than in other regions [1–3], highlighting potential epidemiological characters underlying such clinical benefit.

Previous studies have identified a number of distinct biomarkers to predict the efficacy of ICI treatment [4–24], such as PD-L1 expression, tumour-infiltrating lymphocytes (Tils), Tils derived interferon-γ (IFN-γ), tumour mutational burden (TMB), tumour neoantigen burden (TNB), mismatch repair deficiency (dMMR)/high-level microsatellite instability (MSI-H), oncogenic driver mutations (EGFR, KRAS, and ALK), gut microbiota, peripheral immune cell, circulating tumour DNA, PD-L1-high circulating tumour cell, soluble PD-L1, peripheral cytokines, and patient previous history or pathological features (COPD, smoking, family history of cancer), of which the expression of PD-L1 on tumour cells or tumour-infiltrating immune cells and the tumour mutational burden are the most widely used ones in clinic practice. However, PD-L1 and TMB have been shown to represent independent and uncorrelated predictive variables in multiple studies [17, 19, 25–27]. And the frequency of PD-L1 expression and TMB varied drastically between not only individual tumours but also different tumour types [28–32].

On the other hand, PD-L1 and TMB in the tumour microenvironment, alone or in combination, can categorize tumours into groups as “hypermutated and inflamed”, “hypermutated”, “inflamed”, and “non-hypermutated and non-inflamed”, which might respond differently to ICIs [32, 33]. Therefore, a comprehensive analysis on the prevalence of PD-L1 expression and TMB and their relationship in different tumour types may enlighten the clinical practice of ICI therapies and help to more accurately select ICI responders. Besides, CD8 or PD-1 expression is a sign of the inflammatory response in the tumour microenvironment. A retrospective study reported that the expression of CD8 and PD-1 can also be used to predict the efficacy of ICIs, and the predictions by TMB, PD-1, and CD8 together can be used to explain the objective response rate of most tumour types after receiving ICIs [34]. PD-L1, TMB, PD-1, and CD8 are each a potentially relevant link in the antitumour immune response process; however, their correlations have not been reported with large samples from multiple cancer types. Importantly, the frequency and correlation of these biomarkers in different types of solid tumours from Chinese patients have not been reported in large sample sizes.

In this study, we sought to determine the frequencies of and the correlation between PD-L1 expression and TMB, and their correlations with Tils infiltration in tumour tissue samples from 6668 Chinese advanced tumour patients across multiple tumour types. We also analysed the distribution of these biomarkers and their relationship with the efficacy of anti-PD-1 therapy in a small cohort of NSCLC patients.

## Methods

### Patient recruitment and sample collection

This study included 6668 patients with advanced solid tumours, representing a total of 25 tumour types, who were tested for pharmacodynamics-related biomarkers at the Genecast Precision Medicine Technology Institute between March 2016 and February 2019. Each patient had a sufficient amount of tumour tissue sample for the detection of PD-L1, TMB, and PD-1. The clinical information of the patients is shown in Additional file 1: Table S1. Samples from 347 NSCLC patients were used to study the relationship between CD8 and PD-1, 33 of which received anti-PD-1 therapy. The relationship between biomarkers including PD-L1, TMB, PD-1, and CD8 with the efficacy of anti-PD-1 therapy was evaluated in these 33 cases. This study was conducted with the approval of the ethics committees of the Beijing Ditan Hospital affiliated with the Capital Medical University and the Genecast Precision Medicine Technology Institute. Each patient signed a written informed consent form.

### Immunohistochemical staining for PD-L1, PD-1, MLH1, MSH2, MSH6, and PMS2

Paraffin-embedded tumour tissue samples were sectioned at a thickness of 4 µm and transferred to coated glass slides. For PD-L1 staining, the slides were stained with a Ventana GX automated system (Ventana, AZ, USA). Antigen retrieval was performed in cell conditioning 1. The primary antibody specific for PD-L1 (clone SP142) was diluted 1:25 and incubated for 32 min at room temperature to stain tumour cells. The antibody was detected with the Ventana Amplification Kit and Ventana ultraView Universal DAB Detection Kit. Digital images were captured using an Aperio Scanscope AT Turbo slide scanner under 20× magnification. Scoring of PD-L1 expression was performed using digital image analysis software, namely, Aperio Membrane v9 and Aperio Genie Classifier. PD-L1 expression was reported as a continuous variable of the percentage of tumour cells staining with any intensity. PD-L1 expression for each sample was also classified as negative, low-positive, or high-positive PD-L1 expression. Negative expression was defined as < 1% of tumour cells staining. Low-positive expression was ≥ 1% and < 50% of tumour cells staining. High-positive expression was ≥ 50% of tumour cells staining. These scoring systems were based on previous studies using an SP142 assay [35–37].

For PD-1, MLH1, MSH2, MSH6, and PMS2 staining, high-pressure reparation was used for antigen retrieval. Endogenous peroxidase activity was blocked by 3% hydrogen peroxide for 10 min. After repeated washes in PBS, the slides were incubated with antibodies against PD-1 (clone CAL20, ab237728, Abcam; dilution 1:250), MLH1 (clone ES05, RTU, Dako), MSH2 (Clone FE11, RTU, Dako), MSH6 (Clone EP49, RTU, Dako), and PMS2 (Clone EP51, RTU, Dako) according to the manufacturer’s instructions. The binding of the primary antibodies was visualized by a mouse/rabbit hypersensitive polymer method detection system (PV-8000/6000, Zsbio, Beijing, China) at 37 °C for 30 min. Then, DAB was used for colour development for 6 min. Finally, the tissue sections were counterstained with haematoxylin, dehydrated, and mounted. The immune cell score for PD-1 was reported as a continuous variable of the percentage of the tumour area with any intensity of PD-1 staining. The criterion for dMMR was that one or more of the four proteins MLH1, MSH2, MSH6, and PMS2 did not expressed at all in tumour cells in a section. Two pathologists confirmed the quality and results of the experiment.

### Multiplex immunohistochemical staining for PD-1 and CD8

Paraffin-embedded tissue blocks were serially sectioned into 3 µm sections for the following procedures. The slides were deparaffinized in xylene, rehydrated, and washed in tap water before boiling in Tris–EDTA buffer (pH 9; 643901; Klinipath) for epitope retrieval/microwave treatment (MWT). Endogenous peroxidase and protein blocking were performed using Antibody Diluent/Block (72424205, PerkinElmer) for 10 min at room temperature. The antigens were labelled sequentially. Each round of antigen labelling consisted of three steps: primary antibody incubation, secondary antibody incubation, and tyramide signal amplification (TSA) visualization. The antigen-labelled primary/secondary antibody and TSA complex were removed by MWT with Tris–EDTA buffer (pH 9) at the end of each round, and then the next antigen was labelled. The slides were finished with MWT, counterstained with DAPI for 5 min, and mounted in Antifade Mounting Medium (I0052, NobleRyder). The primary antibodies used were anti-PD-1 antibody (clone CAL20, ab237728, Abcam; dilution 1:100) and anti-CD8 antibody (clone 144B, ab17147, Abcam; dilution 1:25). The primary antibodies were incubated according to the manufacturer’s instructions. Next, incubation with Polymer HRP Rb (PV-6001, Zsbio) or Polymer HRP Ms (PV-6002, Zsbio) was performed at 37 °C for 10 min. TSA visualization was performed with the Opal 7-Color IHC Kit (NEL797B001KT, PerkinElmer) containing the fluorophores DAPI, Opal 690 (PD-1), and Opal 540 (CD8) and the TSA Coumarin System (NEL703001KT; PerkinElmer). The slides were scanned using the PerkinElmer Vectra (Vectra 3.0.5, PerkinElmer). Multispectral images were unmixed using spectral libraries built from images of single-stained tissue samples for each reagent using inForm Advanced Image Analysis software (inForm 2.3.0, PerkinElmer). A selection of 15 representative original multispectral images was used to train the inForm software (tissue segmentation, cell segmentation, phenotyping tool, and positivity score). All of the settings applied to the training images were saved in an algorithm to allow batch analysis of multiple original multispectral images of the same tissue. Two pathologists confirmed the quality and results of the experiment.

### DNA extraction and targeted gene capture sequencing

DNA extraction from the FFPE tumour specimens and targeted gene capture sequencing for the TMB test were performed using the standard protocols mentioned previously [38]. A total of 2 ml of whole blood was collected from each patient, and DNA from the peripheral blood lymphocytes was extracted as a normal control. DNA libraries were captured with a designed Genescope panel of 543 genes (Genecast, Beijing, China) that included tumour-related major genes, covering 1.7 Mb of the genome. The captured samples were subjected to Illumina NovaSeq6000 platform using the pair-end sequencing method.

### Bioinformatics pipeline and tumour mutation burden analysis

The bioinformatics pipeline and tumour mutation burden analyses were performed using the standard protocols mentioned previously [38]. The generated raw sequencing reads were filtered for adapter trimming and quality filtering using Trimmomatic. Obtained reads were aligned to the human genome reference hg19 through Burrows-Wheeler Aligner (BWA v0.7.12). SAM files were converted to sorted BAM files using NovoSort (v3.08.00). Genome Analysis Toolkit (GATK v3.7) was used to do local realignment around potential small insertions or deletions (indels) and base recalibration for next step mutation calling procedures, with duplicated reads removed. Then, we used VarDict (v1.5.1) to detect SNV and small indels, and FreeBayes (v1.1.0-44) was adopted to investigate complex mutations. Paired tumour normal sample calling was processed during the mutation calling. To filter out personal germline mutations, DNA translocation analysis was performed with FusionMap (v8.0.2.32). All base substitutions, short insertions, and deletions were initially recorded before filtering. The generated candidate mutations were annotated using Annovar software tools [39] and subsequently filtered using genomic databases such as Catalogue of Somatic Mutations in Cancer (COSMIC), the Short Genetic Variations database (dbSNP), and the Exome Aggregation Consortium (ExAC).

The TMB was defined as the number of somatic, coding, base substitutions, and indel mutations per megabases of the genome examined. All base substitutions and indels in the coding region of the targeted genes, including synonymous alterations, were initially counted before filtering as described above. Alterations that were predicted to be germline by the somatic—germline zygosity algorithm were not counted. Known germline alterations in dbSNP were not counted. Germline alterations occurring with two or more counts in the ExAC database were not counted [40]. To calculate the TMB per megabases, the total number of mutations counted was divided by the size of the coding region of the targeted territory. TMB was reported as a continuous variable. According to the TMB level, patients were divided into three groups: high, moderate, and low. The grouping criteria were based on the 75th percentile and 25th percentile of this batch of data. Then, a TMB level that was greater than or equal to the 75th percentile was defined as high. A TMB level less than the 25th percentile was defined as low, and the moderate level occurred between the 25th and 75th percentiles.

### Validation of TMB test by whole-exome sequencing (WES)

WES was performed for tumour samples and matched normal control samples from 526 patients on the Illumina NovaSeq6000 platform using the pair-end sequencing method. High-quality paired-end reads were aligned to the hg19 reference genome using the Burrows-Wheeler Aligner (BWA). The VarDict and FreeBayes programs were used for single nucleotide variation (SNV) and indel calling while the ANNOVAR assay was used for the functional annotation of genetic variants. The somatic SNVs and indels were filtered as previously reported [30]. For the determination of TMB, the number of somatic nonsynonymous SNVs in the whole exome (with depth > 40× and allele frequency ≥ 0.05) was quantified. Alterations known to be oncogenic drivers were excluded. TMB was measured by mutations per megabases. The validation result showed that the TMB value from the targeted gene capture sequencing was consistent with the WES-based TMB value (Additional file 2: Figure S1).

### Statistical analyses

Statistical analyses were conducted using GraphPad Prism (version 8.2.0, La Jolla, CA, USA) and SPSS version 22.0 (SPSS, Inc., Chicago, IL, USA). Correlations between two markers were tested using the Spearman or Pearson correlation tests. The nonparametric Mann–Whitney U test was used to test the significance of differences between two populations. The Kruskal–Wallis test was used to test the significance of differences among three populations. All reported *P*-values are two-tailed, and for all analyses, *P *≤ 0.05 was considered significant unless otherwise specified. A heat map and cluster analysis were implemented using R software (v3.5.1) and ComplexHeatmap (bioconductor) package.

## Results

### PD-L1 expression and TMB landscape across tumour types

**Fig. 1:**
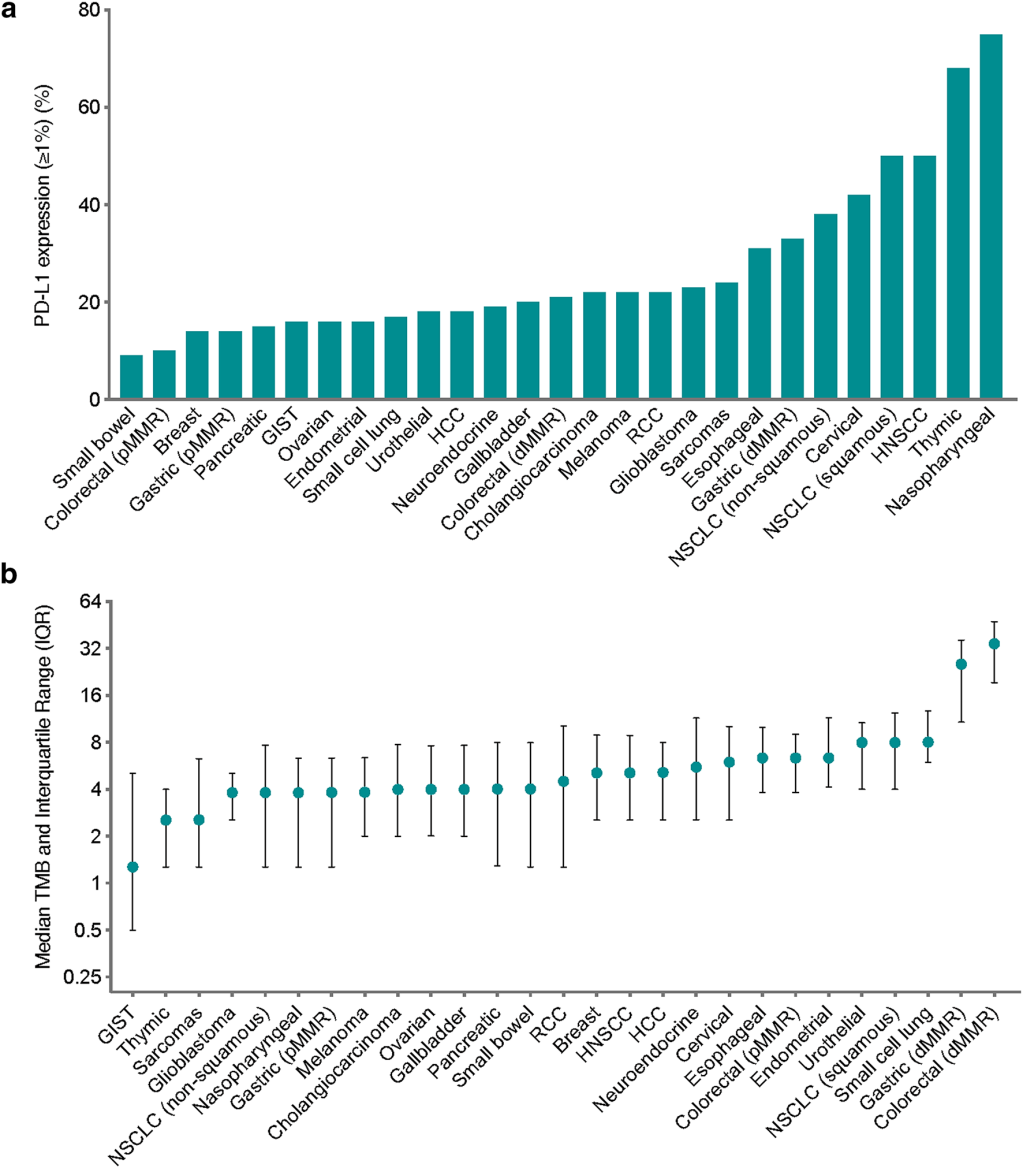
Landscape of PD-L1 expression and TMB across tumour types. **a** Percentage of tumours with positive PD-L1 expression (≥ 1%) by IHC within 25 major tumour types, from the lowest frequency of positivity (left) to the highest frequency (right). **b** Tumour types are ordered from the lowest median TMB (left) to the highest median TMB (right). dMMR, mismatch repair deficient

In total, 6668 patients with advanced tumours representing 25 tumour types were enrolled in the study, and samples with paired PD-L1 expression and TMB value were obtained during the course of standard clinical care. A summary of PD-L1 expression assessed by qualitative immunohistochemical staining is shown in Fig. 1a. PD-L1 expression varied widely among the tumour types examined. Nasopharyngeal carcinoma and thymic carcinoma had the highest frequency of PD-L1 positivity (75% and 68%, respectively), whereas small bowel carcinoma had the lowest frequency of PD-L1 positivity (9%, Fig. 1a). Across all the samples, 3.6% were defined as PD-L1 high-positive (≥ 50% tumour cells stained positive for PD-L1). Strikingly, 51% samples with nasopharyngeal carcinoma and 46% with thymic carcinoma were PD-L1 high-positive, in sharp contrast to 0% with endometrial cancer (Additional file 2: Figure S2).

The median TMB across all samples was 5.08 mutations/Mb (interquartile range: 1.99–8.89). A summary of TMB in different tumour types is shown in Fig. 1b. The median TMBs for each tumour type ranged from 1.27 mutations/Mb in gastrointestinal stromal tumour (GIST) to 34.36 mutations/Mb in dMMR colorectal cancer. dMMR was detected in 21 tumour types (except GIST, neuroendocrine tumour, renal cell carcinoma (RCC) and thymic carcinoma), and tumours with dMMR had higher TMBs, in agreement with the physiological function of the mismatch repair pathway (*P* < 0.0001) (Additional file 2: Figure S3A-B). In addition, small cell lung cancer had the highest median TMB among the non-dMMR cancers (Fig. 1b), suggesting mutations in alternative pathways may associate with high TMB in those tumours. However, overall, PD-L1 expression did not differ between dMMR and pMMR tumours (*P* = 0.779, Additional file 2: Figure S3C).

### Relationship between PD-L1 expression and TMB

**Fig. 2:**
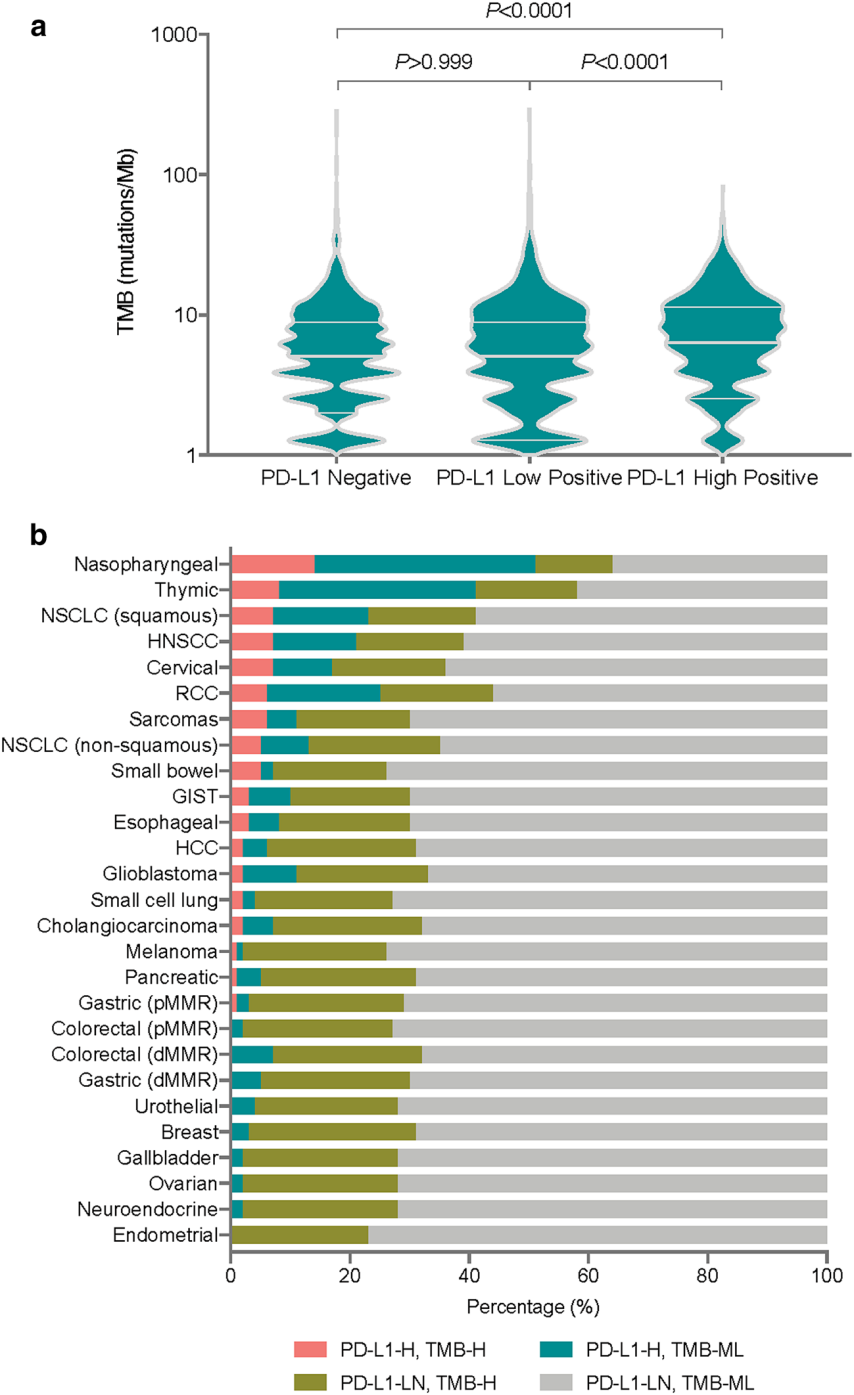
Relationship between TMB and PD-L1 expression and sample classifications. **a** Differences in TMB between PD-L1-negative, low-positive and high-positive tumours. Kruskal–Wallis test. **b** Proportion of samples based on PD-L1-high and TMB-high classifications in different tumour types. PD-L1-H, PD-L1 high-positive. PD-L1-LN, PD-L1 low-positive and negative. TMB-H, TMB high. TMB-ML, TMB moderate and low

We first ranked all the specimens into three grades according to the PD-L1 levels: PD-L1-negative, PD-L1-low-positive, and PD-L1-high-positive (see Methods). PD-L1-high-positive specimens had higher TMBs than PD-L1-low-positive and negative specimens (both *P* < 0.0001, Fig. 2a). Then we assessed the correlation between PD-L1 expression and TMB in each individual tumour type. Across all individual specimens examined, there was a weak positive association between PD-L1 expression and TMB (Spearman R = 0.059, *P* < 0.0001, Additional file 1: Table S2). However, such relationship varied from weak negative in GIST (Spearman R = − 0.1976) to moderately positive in neuroendocrine cancer (Spearman R = 0.3557, Additional file 1: Table S2). The most positive associations between PD-L1 expression and TMB were observed in endometrial and neuroendocrine cancers (Spearman R both > 0.3, and *P* = 0.0332 and *P* = 0.0029, respectively). There were also weak but positive associations between PD-L1 expression and TMB in cervical, pMMR gastric, HNSCC, NSCLC (non-squamous), NSCLC (squamous), and sarcomas (Spearman R all < 0.3, *P* < 0.05). These results may suggest tissue-specific interplay between PD-L1 expression and TMB during tumorigenesis.

All specimens were further classified into four classes based on the level of PD-L1 expression and their TMB: PD-L1 high-positive and TMB high (PD-L1-H, TMB-H), PD-L1 low-positive and negative and TMB high (PD-L1-LN, TMB-H), PD-L1 high-positive and TMB moderate and low (PD-L1-H, TMB-ML), and PD-L1 low-positive and negative and TMB moderate and low (PD-L1-LN, TMB-ML). Figure 2b shows the proportionate of these four sample classes in each tumour types represented in this study. Nasopharyngeal cancer had the highest proportion of samples with both high-positive of PD-L1 and high TMB (14%). However, endometrial, neuroendocrine, ovarian, gallbladder, breast, urothelial, dMMR gastric, dMMR colorectal, and pMMR colorectal cancers had no samples in the PD-L1-H and TMB-H class.

### PD-1^+^ Tils infiltration is correlated with PD-L1 expression, but not to TMB

**Fig. 3:**
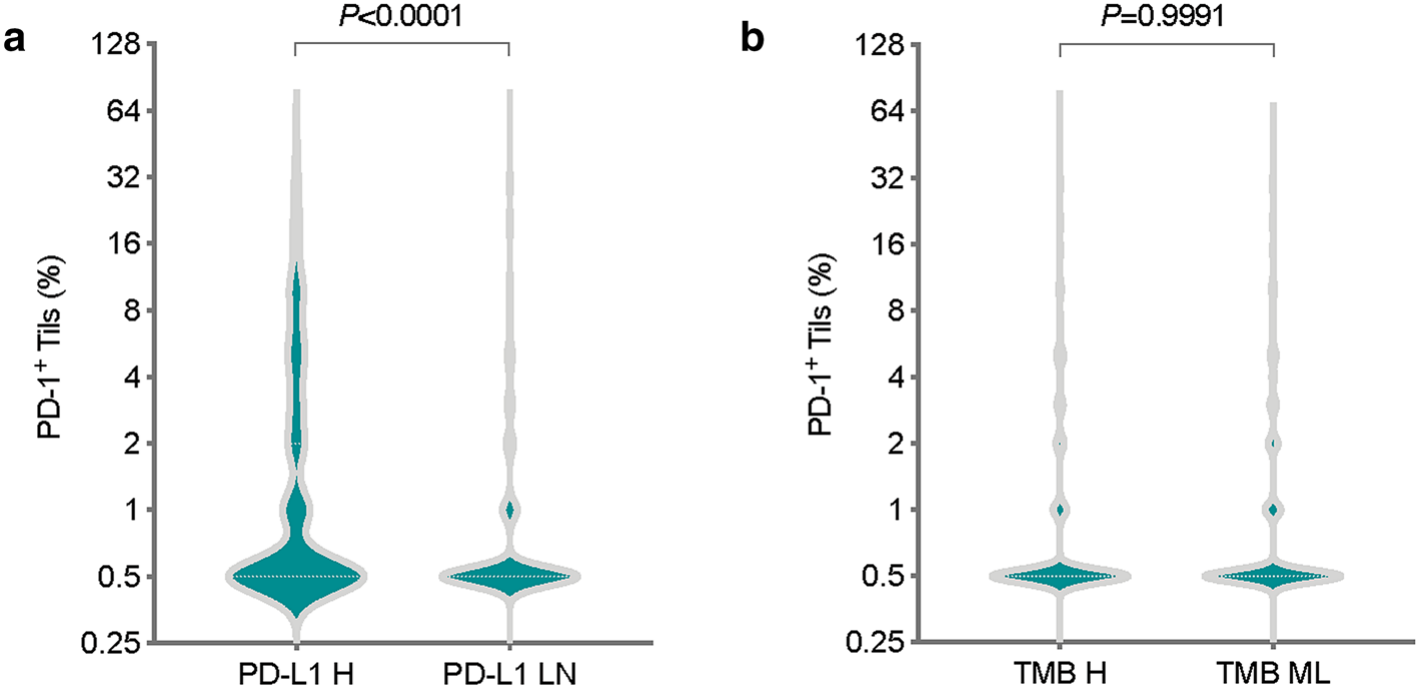
Differences in PD-1^+^ Tils infiltration between groups divided by PD-L1 expression and TMB. **a** Differences in PD-1^**+**^ Tils infiltration between PD-L1-high-positive tumours and PD-L1-negative or low-positive tumours. Mann–Whitney U test. **b** Differences in PD-1^**+**^ Tils infiltration between TMB-high tumours and TMB-moderate or low tumours. Mann–Whitney U test

The correlations of PD-1^+^ Tils infiltration with PD-L1 expression or TMB are shown in Fig. 3. In Fig. 3a, all specimens were separated into PD-L1-negative/low-positive and PD-L1-high-positive groups according to PD-L1 levels. PD-L1-high-positive specimens had a higher PD-1^+^ Tils infiltration rate than PD-L1-negative/low-positive specimens (*P* < 0.0001). Next, the correlations between PD-1 + Tils infiltration and PD-L1 expression were measured in different tumour types. Across all individual samples, there was a positive association between the PD-1^+^ Tils infiltration and the PD-L1 expression (Spearman R = 0.3056, *P* < 0.0001) (Additional file 1: Table S3). Although the relationship between these two biomarkers was not consistent across all tumour types, the strongest association was observed in breast, gallbladder, HNSCC, melanoma, and neuroendocrine cancers (Spearman R all > 0.4). In cervical, dMMR colorectal, endometrial, dMMR gastric, GIST, glioblastoma, small bowel, thymic, and urothelial cancers, the correlations were not significant (Additional file 1: Table S3).

To assess the associations between PD-1^+^ Tils infiltration and TMB, all specimens were separated into TMB-moderate/low and TMB-high groups. Figure 3b shows there was no difference in the PD-1^+^ Tils infiltration between TMB-moderate/low and TMB-high specimens (*P* = 0.9991). There was also no correlation between the PD-1^+^ Tils infiltration and TMB, neither across all individual samples nor across tumour types (Additional file 1: Table S4).

### CD8^+^ T cell infiltration is related to PD-1^+^ Tils infiltration and PD-L1 expression, but not to TMB

**Fig. 4:**
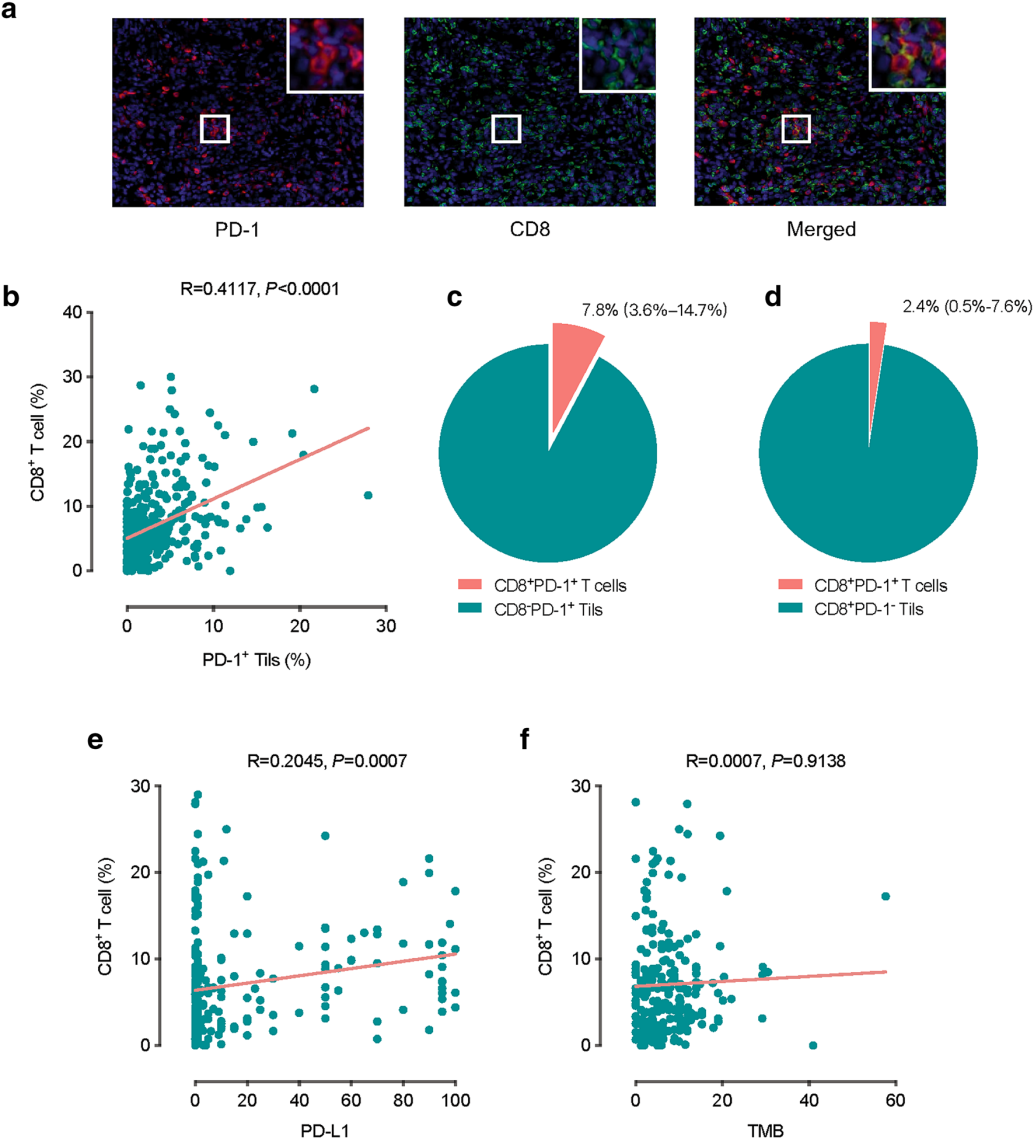
Relationships among CD8^+^ T cell infiltration, PD-1^+^ Tils infiltration, PD-L1, and TMB. **a** Representative images of PD-1^**+**^ Tils, CD8^**+**^ T cells, and CD8^**+**^PD-1^**+**^ T cells in the same slide from a lung adenocarcinoma sample evaluated by multiplex immunohistochemical staining. 20× magnification. **b** Correlation analysis between PD-1^**+**^ Tils and CD8^**+**^ T cells. Spearman correlation test. **c**, **d** The median proportion of CD8^**+**^PD-1^**+**^ T cells in total PD-1^**+**^ Tils and total CD8^**+**^ T cells. In the brackets is the interquartile range (IQR). **e** Correlation analysis between PD-L1 and CD8^**+**^ T cells. Spearman correlation test. **f** Correlation analysis between TMB and CD8^**+**^ T cells. Spearman correlation test

We labelled CD8 and PD-1 proteins on the same slides from 347 NSCLC samples by multiplex immunohistochemistry and analysed the content of CD8^+^ T cells, PD-1^+^ Tils, and CD8^+^PD-1^+^ T cells (Fig. 4a). The frequency of PD-1^+^ Tils, CD8^+^ T cells, and CD8^+^PD-1^+^ T cells across all individual samples varied, and their median frequency was 5.5% (IQR 2.9–8.9%), 1.7% (IQR 0.6–4.2%), and 0.1% (IQR 0–0.5%) respectively. There was a positive association between CD8^+^ T cells and PD-1^+^ Tils (Spearman R = 0.4117, *P *< 0.0001) (Fig. 4b). However, the median proportions of CD8^+^PD-1^+^ T cells in total PD-1^+^ Tils and total CD8^+^ T cells were not high, 7.8% (IQR 3.6–14.7%) and 2.4% (IQR 0.5–7.6%), respectively (Fig. 4c, d). Further analysis also found a positive association between CD8^+^ T cells and PD-L1 expression (Spearman R = 0.2045, *P *= 0.0007), but there was no correlation between CD8^+^ T cells and TMB (Spearman R = 0.0007, *P *= 0.9138) (Fig. 4e, f).

### PD-L1 expression, TMB, PD-1^+^ Tils, and CD8^+^ T cell infiltration are related to the response to anti-PD-1 therapy in NSCLC

**Fig. 5:**
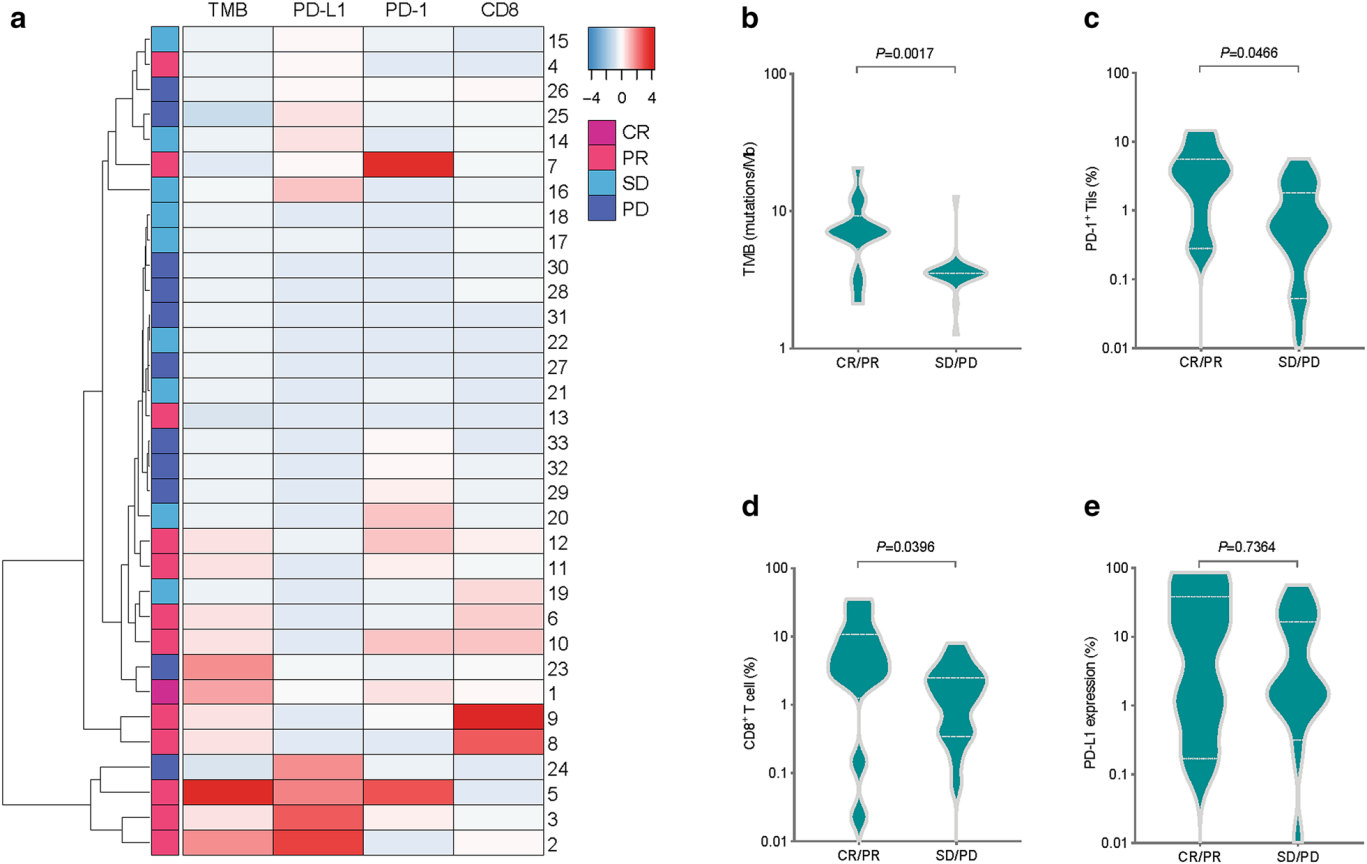
Relationships among PD-L1, TMB, PD-1^+^ Tils, CD8^+^ T cell, and response to anti-PD-1 therapy. **a** Heatmap result of the PD-L1 expression, TMB, PD-1^**+**^ Tils infiltration, and CD8^**+**^ T cell infiltration in different groups according to the response to anti-PD-1 therapy. **b**–**e** Differences in PD-L1 expression, TMB, PD-1^**+**^ Tils infiltration, and CD8^**+**^ T cell infiltration between the CR/PR group and SD/PD group. Mann–Whitney U test. *CR* complete response, *PR* partial response, *SD* stable disease, *PD* progressive disease

In the 347 NSCLC patients mentioned above, 33 of them received anti-PD-1 therapy or anti-PD-1 therapy plus chemotherapy. All patients were EGFR/KRAS wild-type, 14 cases of which were lung adenocarcinoma and 20 cases lung squamous cell carcinoma. 10 patients received PD-1 inhibitor monotherapy and 23 received a combination of PD-1 inhibitor plus chemotherapy. Detailed clinical information on those cases is provided in Additional file 1: Table S5. The efficacy of treatment was evaluated as objective tumour response: 1 case achieved CR (complete response); 12 cases achieved PR (partial response); 9 cases had SD (stable disease); and 11 patients had PD (progressive disease). There was no significant difference in objective response rates (ORR = (CR + PR)/(CR + PR + SD + PD)) between the PD-1 inhibitor monotherapy group and the PD-1 inhibitor plus chemotherapy group. The ORR of the monotherapy group was 40.0%, and 39.1% of the PD-1 inhibitor plus chemotherapy group. The CR/PR group had higher levels of PD-L1 expression, TMB, PD-1^+^ Tils infiltration, and CD8^+^ T cell infiltration, and most patients in this group exhibited concomitantly elevated levels of multiple biomarkers (Fig. 5a). These four markers were at lower levels in the SD/PD group (Fig. 5a). Generally, the levels of three biomarkers, TMB, PD-1^+^ Tils infiltration, and CD8^+^ T cell infiltration, were significantly higher in the CR/PR group than in the SD/PD group (*P *= 0.0017, *P *= 0.0466, and *P *= 0.0396, respectively) (Fig. 5b–d). The PD-L1 expression was also higher in the CR/PR group than in the SD/PD group, but this difference was not significant (*P *= 0.7364) (Fig. 5e).

## Discussion

To the best of our knowledge, this is the largest report on the PD-L1 expression by IHC and TMB by targeted gene capture sequencing across multiple tumour types from Chinese advanced cancer patients. We found that PD-L1 expression and TMB varied widely among the tumour types (Fig. 1). This result is similar to the previous report in general [32]. However, in some tumour types, the status of PD-L1 expression and TMB differed from our findings. Our study found that the PD-L1 expression and TMB levels of melanoma were lower than those reported in previous studies [32]. This may be due to differences in the tissue origin of this tumour. Chinese melanoma is mostly the mucosal type while malignant melanomas in Europe and the United States are mostly the skin type, the onset of which is usually the result of accumulation of genomic mutations caused by UV. Accordingly, previous studies have confirmed a higher TMB in skin type malignant melanoma [41, 42]. A Chinese tumour patient study as well as a TCGA database study showed that gastric cancer and colorectal cancer also have relatively higher TMBs [41, 43], but these studies have not fully demonstrated the impact of dMMR/MSI-H on TMB. In order to provide more accurate information on TMB in different tumour types, our study specifically separated dMMR gastric and colorectal cancers from non-dMMR types and observed much higher TMBs in the former types. In oesophageal cancer, the positive rate of PD-L1 was much higher than that in previous reports. Recent studies have found that oesophageal cancers in China have a better efficacy after ICI treatment than in other regions [1]. These results suggest that specialized studies of ICI-related biomarkers in different populations are needed.

The independence between PD-L1 expression and TMB within most tumour types suggests that each biomarker could independently inform the use of ICI therapy in tumours with different microenvironments. Previous reports using these two biomarkers defined the immunologic states of the tumour microenvironment as “hypermutated and inflamed”, “hypermutated”, “inflamed”, or “non-hypermutated and non-inflamed” [32, 33]. The non-hypermutated and non-inflamed type of tumour may be resistant to ICI monotherapy, while the hypermutated and inflamed types of tumour may stand the best chance of benefiting from the ICI monotherapy. Based on our results in this study, some tumour types, such as nasopharyngeal, NSCLC, and HNSCC, can be classified in the same way. However, it is not applicable to other tumour types, such as endometrial, breast, urothelial, colorectal, etc. (Figure 2), in which no or few hypermutated and inflamed samples were observed. Thus, the efficacy of ICI monotherapy on these tumour types require validations by clinical trials. The immunotherapeutic intervention strategy that combines other treatments also needs to be considered, and biomarkers associated with their efficacy may need to be evaluated from other perspectives.

A previous study in lung cancer revealed a weak correlation between Tils with PD-L1 expression, but not with TMB [44]. While the sample size in this study was small, our study assessed the correlations of these three biomarkers in a larger size of sample with lung cancer. We found that the level of CD8^+^ T cells was weakly correlated with the expression of PD-L1, but not with TMB, which is in agreement with the report mentioned above. A study based on the TCGA database showed that high level of RNA expression of PD-L1 and CD8A was significantly associated with a high number of neoantigens [42]. However, our results showed that the TMB, which was considered closely associated with the neoantigen, was not very related to PD-L1 or CD8 expression measured by IHC. We considered that the difference in methodologies may be the major cause of this discrepancy: While the TCGA study used RNA sequencing, our study used IHC to quantify the expression levels of PD-L1 and CD8. We further verified the independence of PD-L1, CD8, and TMB in a small cohort of NSCLC received ICI therapy, which provided additional information on application examples.

PD-1 expression has also shown predictive power in evaluating the efficacy of ICI therapy [34, 45]. Our study found that in most tumour types there was a positive association between the PD-1^+^ Tils infiltration and the PD-L1 expression (Fig. 3 and Additional file 1: Table S3); in addition, PD-1^+^ Tils did correlate with CD8^+^ T cells in the NSCLC cohort (Fig. 4). In T cells, PD-1 expression may indicate cell activation. Similar to PD-1, PD-L1 expression can also be a marker of immune activation. The expression of PD-L1 on tumours and in the tumour microenvironment is mostly dependent on the immune activation pathway of IFN-γ. IFN-γ produced by effector T cells soon after but not before activation of immune response is the major inducer of PD-L1 expression at the transcription level [46, 47]. However, PD-1 is generally expressed on many types of Tils in tumour tissues and not only on effector T cells. Our study found that the proportion of CD8^+^PD-1^+^ T cells in the total PD-1^+^ Tils varied greatly in different samples, but overall, it was not high (median 7.8% (IQR 3.6%-14.7%)) (Fig. 4). Other immune cells in large amounts in the microenvironment can also express PD-1. We speculate that the efficacy of ICI treatment may not be good when there are a large number of Tregs or MDSCs in the tumour microenvironment. This hypothesis requires further research to provide practical evidence. On the other hand, Tils in the tumour microenvironment, especially CD8^+^ T cells, also express a large number of other immune checkpoint molecules, such as LAG-3, TIM-3, TIGIT, VISTA, B7-H3, and BTLA [48]. These molecules will also seriously damage the tumour killing by T cells. Theoretically, the efficiency of combined blocking PD-1 and other checkpoint molecules may be better than the anti-PD-1 mono-treatment.

In this study, we found that PD-1^+^ Tils infiltration did not correlate with TMB (Fig. 3 and Additional file 1: Table S4). Interestingly, recent studies have shown that the efficacy of anti-PD-1 therapy is not significantly associated with TMB [49–51]. Our results provide an explanation for the outcomes of these clinical trials that TMB-H tumours may not have enough drug-reactive PD-1^+^ Tils to elicit a therapeutic response to PD-1 inhibitors during initial treatment. It is also worth noting although TMB is a widely used biomarker for patient selection and efficacy prediction, it is not an ideal replacement for immunogenic neoantigens. High TMB can increase the possibility of generating immunogenic neoantigens but cannot guarantee the occurrence of specific immune reactions to neoantigens [24]. Cancer cells with strong immunogenicity are easily eliminated by cancer immunoediting, while subpopulations with weak immunogenicity can survive. As a result, some cancer cells could escape immune attack and evolve into obvious clinical lesions. Recently, promoter hypermethylation of neoantigen genes has been proposed to be a vital mechanism of immunoediting. Rosenthal et al. found that the hypermethylation of promoters of neoantigen genes contributed to the decreased cancer immunogenicity [52, 53]. Their further investigation revealed that the proportion of ubiquitously expressed clonal neoantigens was significantly decreased in tumours with abundant Tils. At the transcriptional level, neoantigen transcripts were depleted by such immune pressure. This suggests that when applying TMB to patient selection or response prediction, its complex relationship with neoantigen should be considered from all aspects.

A retrospective study reported that TMB, PD-1, and CD8 together can explain the objective response rate of most tumour types after receiving ICIs [34]. We found a similar phenomenon in this study. In the NSCLC cases receiving anti-PD-1 therapy, the CR/PR group had higher levels of PD-L1 expression, TMB, PD-1^+^ Tils infiltration, and CD8^+^ T cell infiltration (Fig. 5), and most cases in this group have high levels of multiple biomarkers at the same time. Some of these samples could be listed as “hypermutated and inflamed”, “hypermutated”, or “inflamed” types as mentioned above. However, there was no such phenomenon in the SD/PD group, and most of the samples in this group could be categorized into the “non-hypermutated and non-inflamed” type. We believe that the classification of “hypermutated and inflamed”, “hypermutated”, or “inflamed” should not be based only on the levels of TMB and PD-L1 but rather the combinatorial assessment of various biomarkers such as TMB, PD-L1, PD-1, and CD8, which may be more effective in helping to find more responders to PD-1 inhibitor therapy. In addition, several other predictive markers were investigated recently including dMMR/MSI-H, oncogenic driver mutations (EGFR, KRAS, and ALK), gut microbiota, peripheral immune cell, circulating tumour DNA, peripheral cytokines, and patient previous history or pathological features (COPD, smoking, and family history of cancer) [23]. In sum, establishing a comprehensive assessment framework involving multiple biomarkers will be meaningful for studying the immune status of tumours and selecting patients who will be sensitive to anti-PD-1/PD-L1 therapy.

The advantages of this study include the use of clinically validated analytical methods in a CAP-certified laboratory to report PD-L1 expression, TMB, and other markers in a large number of representative clinical samples from Chinese patients. A limitation is that the sample used to observe the therapeutic relationship between biomarkers and ICI was derived from only one tumour type, and the sample size was small. The role of these markers in the treatment of ICI in different tumour types requires further exploration and validation.

## Conclusions

In summary, we analysed PD-L1 expression, TMB, and Tils infiltration and their correlations in various types of advanced solid tumours from Chinese patients. These data may inform ICI treatment and help to identify the type of tumour or individual patient that are most likely or least likely to benefit from ICI treatment.

## Supplementary information

**Additional file 1: Table S1.** Patient’s clinical characteristics. **Table S2.** Relationship between PD-L1 expression and TMB across tumour types. **Table S3.** Relationship between PD-1^+^ Tils infiltration and PD-L1 expression across tumour types. **Table S4.** Relationship between PD-1^+^ Tils infiltration and TMB across tumour types. **Table S5.** Clinical information of 33 cases of NSCLC patients who have received anti-PD-1 therapy.

**Additional file 2: Figure S1.** Validation of TMB by whole-exome sequencing (WES). **Figure S2.** Landscape of PD-L1 high-positive samples across tumour types. **Figure S3.** dMMR across tumour types and its relationships with TMB and PD-L1 expression.

## Data Availability

Not applicable.

## Abbreviations

PD-L1: Programmed death ligand-1
PD-1: Programmed death-1
TMB: Tumour mutational burden
ICI: Immune checkpoint inhibitor
dMMR: Mismatch repair deficiency
pMMR: MMR-proficient
NGS: Next generation sequencing
WES: Whole-exome sequencing
Tils: Tumour-infiltrating lymphocytes
CR: Complete response
PR: Partial response
SD: Stable disease
PD: Progressive disease
