# Supplementary material for: The frequency and inter-relationship of PD-L1 expression and tumour mutational burden across multiple types of advanced solid tumours in China
Source: Exp Hematol Oncol. 2020 Aug 3;9:17. doi: 10.1186/s40164-020-00173-3 (PMC7397649; doi:10.1186/s40164-020-00173-3)
Supplement: Supplementary file 1 — Additional file 1: Table S1. Patient’s clinical characteristics. Table S2. Relationship between PD-L1 expression and TMB across tumour types. Table S3. Relationship between PD-1+ Tils infiltration and PD-L1 expression across tumour types. Table S4. Relationship between PD-1+ Tils infiltration and TMB across tumour types. Table S5. Clinical information of 33 cases of NSCLC patients who have received anti-PD-1 therapy. [file 40164_2020_173_MOESM1_ESM.docx]

**Additional tables**

**Additional Table S1. Patient's clinical characteristics.**

| Tumour type | Number of cases | Age | Gender | | Clinical stage | |
| --- | --- | --- | --- | --- | --- | --- |
|  |  | Median  (range) | Male  n (%) | Female  n (%) | IIIB  n (%) | IV  n (%) |
| Breast | 127 | 50 (43-56) | 0 (0) | 127 (100) | 49 (39) | 78 (61) |
| Cervical | 73 | 49 (43-56) | 0 (0) | 73 (100) | 17 (23) | 56 (77) |
| Cholangiocarcinoma | 117 | 60 (53-65) | 69 (59) | 48 (41) | 56 (48) | 61 (52) |
| Colorectal | 759 | 58 (48-68) | 459 (60) | 300 (40) | 469 (62) | 290 (38) |
| Endometrial | 43 | 58 (52-63) | 0 (0) | 43 (100) | 11 (26) | 32 (74) |
| Esophageal | 161 | 63 (55-69) | 141 (88) | 20 (12) | 43 (27) | 118 (73) |
| Gallbladder | 50 | 63 (53-68) | 20 (40) | 30 (60) | 17 (34) | 33 (66) |
| Gastric | 478 | 58 (48-66) | 307 (64) | 171 (36) | 95 (20) | 383 (80) |
| GIST | 30 | 58 (49-67) | 14 (47) | 16 (53) | 10 (33) | 20 (67) |
| Glioblastoma | 46 | 47 (30-54) | 28 (61) | 18 (39) | 43 (93) | 3 (7) |
| HCC | 183 | 54 (47-63) | 149 (81) | 34 (19) | 34 (19) | 149 (81) |
| HNSCC | 152 | 60 (50-67) | 98 (64) | 54 (36) | 34 (22) | 118 (78) |
| Melanoma | 74 | 50 (44-65) | 29 (39) | 45 (61) | 23 (31) | 51 (69) |
| Nasopharyngeal | 80 | 50 (43-59) | 65 (81) | 15 (19) | 32 (40) | 48 (60) |
| Neuroendocrine | 68 | 58 (48-66) | 49 (72) | 19 (28) | 21 (31) | 47 (69) |
| NSCLC (non-squamous) | 2835 | 60 (52-66) | 1541 (54) | 1294 (46) | 1080 (38) | 1755 (62) |
| NSCLC (squamous) | 699 | 64 (56-69) | 609 (87) | 90 (13) | 225 (32) | 474 (68) |
| Ovarian | 102 | 52 (44-61) | 0 (0) | 102 (100) | 30 (29) | 72 (71) |
| Pancreatic | 186 | 61 (55-66) | 125 (67) | 61 (33) | 46 (25) | 140 (75) |
| RCC | 32 | 56 (44-68) | 21 (66) | 11 (34) | 9 (28) | 23 (72) |
| Sarcomas | 141 | 49 (35-63) | 58 (41) | 83 (59) | 25 (18) | 116 (82) |
| Small bowel | 43 | 60 (52-67) | 18 (42) | 25 (58) | 20 (47) | 23 (53) |
| Small cell lung | 107 | 62 (55-69) | 89 (83) | 18 (17) | 41 (38) | 66 (62) |
| Thymic | 36 | 55 (44-61) | 25 (69) | 11 (31) | 17 (47) | 19 (53) |
| Urothelial | 46 | 66 (59-72) | 29 (63) | 17 (37) | 15 (33) | 31 (67) |
| All samples | 6668 | 59 (51-66) | 3953 (59) | 2715 (41) | 2462 (37) | 4206 (63) |

GIST, gastrointestinal stromal tumour. HCC, hepatocellular carcinoma. HNSCC, head and neck squanlous cell carcinoma. NSCLC, non-small cell lung cancer. RCC, renal cell carcinoma.

**Additional Table S2. Relationship between PD-L1 expression and TMB across tumour types.**

| Tumour type | Number of cases | Spearman R | Spearman P |
| --- | --- | --- | --- |
| All samples | 6668 | 0.05991 | <0.0001 |
| Breast | 127 | -0.1073 | 0.2299 |
| Cervical | 73 | 0.283 | 0.0152 |
| Cholangiocarcinoma | 117 | -0.03967 | 0.6711 |
| Colorectal (dMMR) | 44 | 0.04944 | 0.7499 |
| Colorectal (pMMR) | 715 | 0.04029 | 0.282 |
| Endometrial | 43 | 0.3254 | 0.0332 |
| Esophageal | 161 | 0.05127 | 0.5183 |
| Gallbladder | 50 | 0.08033 | 0.5792 |
| Gastric (dMMR) | 20 | 0.1452 | 0.5415 |
| Gastric (pMMR) | 458 | 0.11 | 0.0186 |
| GIST | 30 | -0.1976 | 0.2953 |
| Glioblastoma | 46 | 0.02971 | 0.8446 |
| HCC | 183 | 0.03108 | 0.6762 |
| HNSCC | 152 | 0.1794 | 0.027 |
| Melanoma | 74 | 0.2059 | 0.0784 |
| Nasopharyngeal | 80 | -0.0117 | 0.918 |
| Neuroendocrine | 68 | 0.3557 | 0.0029 |
| NSCLC (non-squamous) | 2835 | 0.07504 | <0.0001 |
| NSCLC (squamous) | 699 | 0.08979 | 0.0176 |
| Ovarian | 102 | 0.07374 | 0.4614 |
| Pancreatic | 186 | -0.002713 | 0.9707 |
| RCC | 32 | -0.05555 | 0.7627 |
| Sarcomas | 141 | 0.2433 | 0.0037 |
| Small bowel | 43 | 0.1825 | 0.2416 |
| Small cell lung | 107 | 0.04555 | 0.6413 |
| Thymic | 36 | -0.1342 | 0.4353 |
| Urothelial | 46 | 0.1389 | 0.3574 |

Spearman’s correlations.

**Additional Table S3. Relationship between PD-1^+^ Tils infiltration and PD-L1 expression across tumour types.**

| Tumour type | Number of cases | Spearman R | Spearman P |
| --- | --- | --- | --- |
| All samples | 6668 | 0.3056 | <0.0001 |
| Breast | 127 | 0.4269 | <0.0001 |
| Cervical | 73 | 0.2233 | 0.0576 |
| Cholangiocarcinoma | 117 | 0.2487 | 0.0025 |
| Colorectal (dMMR) | 44 | 0.2213 | 0.3626 |
| Colorectal (pMMR) | 715 | 0.2327 | <0.0001 |
| Endometrial | 43 | 0.1014 | 0.5334 |
| Esophageal | 161 | 0.2167 | 0.0116 |
| Gallbladder | 50 | 0.5146 | 0.0026 |
| Gastric (dMMR) | 20 | 0.2031 | 0.4548 |
| Gastric (pMMR) | 458 | 0.2732 | <0.0001 |
| GIST | 30 | -0.4714 | 0.4286 |
| Glioblastoma | 46 | 0.077 | 0.6859 |
| HCC | 183 | 0.3994 | <0.0001 |
| HNSCC | 152 | 0.426 | <0.0001 |
| Melanoma | 74 | 0.465 | <0.0001 |
| Nasopharyngeal | 80 | 0.3163 | 0.0012 |
| Neuroendocrine | 68 | 0.4834 | 0.0003 |
| NSCLC (non-squamous) | 2835 | 0.2788 | <0.0001 |
| NSCLC (squamous) | 699 | 0.2472 | <0.0001 |
| Ovarian | 102 | 0.3367 | 0.0011 |
| Pancreatic | 186 | 0.3619 | <0.0001 |
| RCC | 32 | 0.3991 | 0.0434 |
| Sarcomas | 141 | 0.3535 | 0.0004 |
| Small bowel | 43 | 0.2375 | 0.2063 |
| Small cell lung | 107 | 0.2673 | 0.0042 |
| Thymic | 36 | 0.0835 | 0.6851 |
| Urothelial | 46 | -0.1314 | 0.4127 |

Spearman’s correlations.

**Additional Table S4. Relationship between PD-1^+^ Tils infiltration and TMB across tumour types.**

| Tumour type | Number of cases | Spearman R | Spearman P |
| --- | --- | --- | --- |
| All samples | 6668 | 0.01523 | 0.3894 |
| Breast | 127 | -0.1061 | 0.4238 |
| Cervical | 73 | 0.1475 | 0.3394 |
| Cholangiocarcinoma | 117 | 0.0184 | 0.8881 |
| Colorectal (dMMR) | 44 | 0.09661 | 0.7635 |
| Colorectal (pMMR) | 715 | -0.01711 | 0.7721 |
| Endometrial | 43 | 0.09353 | 0.6712 |
| Esophageal | 161 | 0.2218 | 0.0593 |
| Gallbladder | 50 | 0.4113 | 0.0899 |
| Gastric (dMMR) | 20 | 0.2606 | 0.3469 |
| Gastric (pMMR) | 458 | 0.04787 | 0.5304 |
| GIST | 30 | 0.3961 | 0.5 |
| Glioblastoma | 46 | 0.09759 | >0.9999 |
| HCC | 183 | -0.01492 | 0.8942 |
| HNSCC | 152 | 0.01054 | 0.932 |
| Melanoma | 74 | 0.1615 | 0.2727 |
| Nasopharyngeal | 80 | 0.0393 | 0.7954 |
| Neuroendocrine | 68 | 0.2335 | 0.2228 |
| NSCLC (non-squamous) | 2835 | -0.04248 | 0.1298 |
| NSCLC (squamous) | 699 | -0.07199 | 0.1647 |
| Ovarian | 102 | 0.1584 | 0.2151 |
| Pancreatic | 186 | 0.06672 | 0.4927 |
| RCC | 32 | 0.2216 | 0.4853 |
| Sarcomas | 141 | 0.0865 | 0.4764 |
| Small bowel | 43 | 0.2848 | 0.252 |
| Small cell lung | 107 | 0.2411 | 0.0569 |
| Thymic | 36 | 0.148 | 0.6363 |
| Urothelial | 46 | 0.3889 | 0.0815 |

Spearman’s correlations.

**Additional Table S5. Clinical information of 33 cases of NSCLC patients who have received anti-PD-1 therapy.**

| Case | Gender | Age | Clinical stage | Pathological type | EGFR/KRAS mutation | Intervention | Objective response |
| --- | --- | --- | --- | --- | --- | --- | --- |
| 1 | Male | 68 | IV | ADC | no | Anti-PD1 | CR |
| 2 | Male | 69 | IV | SQCC | no | Anti-PD1+Chemo | PR |
| 3 | Male | 62 | IV | SQCC | no | Anti-PD1 | PR |
| 4 | Male | 61 | IV | SQCC | no | Anti-PD1+Chemo | PR |
| 5 | Male | 64 | IV | SQCC | no | Anti-PD1 | PR |
| 6 | Male | 58 | IIIB | ADC | no | Anti-PD1+Chemo | PR |
| 7 | Male | 60 | IV | SQCC | no | Anti-PD1+Chemo | PR |
| 8 | Male | 65 | IIIB | ADC | no | Anti-PD1+Chemo | PR |
| 9 | Male | 69 | IV | SQCC | no | Anti-PD1 | PR |
| 10 | Male | 65 | IIIB | SQCC | no | Anti-PD1+Chemo | PR |
| 11 | Male | 64 | IIIB | SQCC | no | Anti-PD1+Chemo | PR |
| 12 | Male | 63 | IIIB | SQCC | no | Anti-PD1+Chemo | PR |
| 13 | Male | 49 | IV | SQCC | no | Anti-PD1+Chemo | PR |
| 14 | Male | 50 | IV | SQCC | no | Anti-PD1+Chemo | SD |
| 15 | Male | 47 | IIIB | SQCC | no | Anti-PD1+Chemo | SD |
| 16 | Male | 55 | IV | SQCC | no | Anti-PD1 | SD |
| 17 | Male | 64 | IV | ADC | no | Anti-PD1+Chemo | SD |
| 18 | Male | 55 | IIIB | ADC | no | Anti-PD1+Chemo | SD |
| 19 | Male | 64 | IIIB | ADC | no | Anti-PD1+Chemo | SD |
| 20 | Male | 62 | IV | ADC | no | Anti-PD1+Chemo | SD |
| 21 | Female | 78 | IV | ADC | no | Anti-PD1+Chemo | SD |
| 22 | Male | 54 | IV | SQCC | no | Anti-PD1+Chemo | SD |
| 23 | Male | 55 | IV | SQCC | no | Anti-PD1 | PD |
| 24 | Male | 65 | IV | SQCC | no | Anti-PD1 | PD |
| 25 | Male | 58 | IV | ADC | no | Anti-PD1+Chemo | PD |
| 26 | Male | 65 | IV | ADC | no | Anti-PD1+Chemo | PD |
| 27 | Female | 64 | IV | SQCC | no | Anti-PD1 | PD |
| 28 | Male | 47 | IIIB | SQCC | no | Anti-PD1+Chemo | PD |
| 29 | Male | 60 | IV | ADC | no | Anti-PD1+Chemo | PD |
| 30 | Male | 57 | IV | ADC | no | Anti-PD1+Chemo | PD |
| 31 | Male | 70 | IV | ADC | no | Anti-PD1 | PD |
| 32 | Male | 55 | IV | ADC | no | Anti-PD1 | PD |
| 33 | Female | 41 | IIIB | SQCC | no | Anti-PD1+Chemo | PD |

LAD, lung adenocarcinoma. LSQC, lung squamous cell carcinoma. Chemo, chemotherapy. CR, complete response. PR, partial response. SD, stable disease. PD, progressive disease.
