# Supplementary material for: The frequency and inter-relationship of PD-L1 expression and tumour mutational burden across multiple types of advanced solid tumours in China
Source: Exp Hematol Oncol. 2020 Aug 3;9:17. doi: 10.1186/s40164-020-00173-3 (PMC7397649; doi:10.1186/s40164-020-00173-3)
Supplement: Supplementary file 2 — Additional file 2: Figure S1. Validation of TMB by whole-exome sequencing (WES). Figure S2. Landscape of PD-L1 high-positive samples across tumour types. Figure S3. dMMR across tumour types and its relationships with TMB and PD-L1 expression. [file 40164_2020_173_MOESM2_ESM.docx]

**Additional figures**

- Figure legends of Additional figures.
- Additional Figure S1.
- Additional Figure S2.
- Additional Figure S3.

**Figure legends of additional figures**

**Additional Figure S1. Validation of TMB by whole-exome sequencing (WES).**

Correlation analysis of TMB values quantified by targeted gene capture sequencing and by WES. Pearson correlation test.

**Additional Figure S2. Landscape of PD-L1 high-positive samples across tumour types.**

Percentage of tumours with positive PD-L1 expression (≥ 50%) by IHC within 25 tumour types, from the lowest frequency of positivity (left) to the highest frequency (right).

**Additional Figure S3. dMMR across tumour types and its relationships with TMB and PD-L1 expression.**

A. Percentage of tumours with dMMR by IHC within 25 tumour types, from the lowest frequency of positivity (left) to the highest frequency (right). B. Differences in TMB between dMMR and pMMR tumours. Mann-Whitney U test. C. Differences in PD-L1 expression between dMMR and pMMR tumours. Mann-Whitney U test.

Additional Figure S1

Additional Figure S2

Additional Figure S3
